# Suicide Patterns in Malta: pathways for prevention

**DOI:** 10.1093/eurpub/ckac129.529

**Published:** 2022-10-25

**Authors:** J Cachia

**Affiliations:** Office of the Superintendent of Public Health, Ministry for Health, Valletta, Malta

## Abstract

**Background:**

Suicidal behaviour is the result of several risk factors, such as acute stress, severe depression, violence, sexual abuse, etc. A mental public health approach to suicide prevention needs to look beyond the demographic characteristics of deaths by suicide and to take into account specific country determinants. Available clinical information can help identify and quantify risk, analyse patterns of behaviour, explore links between risk and behaviour and generate possible suicide prevention pathways.

**Methods:**

162 deaths by suicide for the period January 2015 - June 2021 in Malta are analysed against available clinical information. Major sources of detailed clinical information include obligatory notifications to the Commissioner for Mental Health of all cases of persons involuntarily admitted to acute psychiatric services and other medical records held within the Maltese public mental health system.

**Results:**

81% of 162 deaths by suicide for the period under study were males, two-thirds of them between 25 and 54 years. The preliminary findings have confirmed that less than 50% of these deaths had previous contact with the public health system. The two main diagnostic criteria among deaths with recent psychiatric admission/s were acute stress reaction to personal life events and very severe mood disturbances, particularly depression. There seems to be increased risk among migrants and foreign workers residing and working in Malta. The frequency of prior admissions, age-related issues, the time-event relationships, and relevance of elicited clinical findings are still being evaluated and will be presented in more detail in the workshop.

**Conclusions:**

Exploring risk factors within the history of cases of suicide through available clinical information can contribute to the development of suicide prevention pathways, relevant to the specific context of local communities.

